# Occupational post‐exposure prophylaxis among healthcare workers: a scoping review of factors affecting optimal utilization

**DOI:** 10.1002/jia2.26341

**Published:** 2024-08-18

**Authors:** Judith D. Auerbach, Siobhan Malone, Andrew D. Forsyth

**Affiliations:** ^1^ Department of Medicine University of California San Francisco San Francisco California USA; ^2^ Bill & Melinda Gates Foundation Seattle Washington USA; ^3^ Andrew Forsyth Consulting Berkeley California USA

**Keywords:** HIV prevention, post‐exposure prophylaxis, PEP, healthcare workers, occupational exposure, LMIC

## Abstract

**Introduction:**

Post‐exposure prophylaxis (PEP) is an efficacious prevention method when initiated promptly after an HIV exposure. Yet, PEP has been underutilized, even among healthcare workers (HCWs) with occupational exposure in sites with PEP policies and procedures and access to PEP medications. It is important to understand the dynamics of uneven PEP use in what appears to be an optimal context to better protect the health and wellbeing of HCWs.

**Methods:**

We conducted a scoping review to elucidate factors influencing HCWs’ use of PEP after occupational exposure. We searched PubMed, PsychInfo and Google Scholar for peer‐reviewed literature published in English from 2014 to 2022 using the terms *HIV, postexposure/post‐exposure prophylaxis, acceptability*, *healthcare workers*, and *values and preferences*. An inductive narrative review of the resulting 53 studies identified core themes.

**Results:**

Nearly all studies (96%) with various HCW types and settings occurred in low‐ and middle‐income countries (LMICs) in Africa and Asia. Identified themes arrayed along a trajectory of PEP use experience: awareness/knowledge; acceptability; availability/access; uptake/use; adherence/completion. Across studies, awareness of PEP for HIV prevention was high, knowledge about drug regimens and healthcare facility policies was moderate to low; acceptability of PEP was moderate to high; PEP's perceived accessibility/availability was inconsistent and varied by geographic location and setting; HCWs’ uptake of PEP was low, affected by not knowing how to report an exposure and being unaware of PEP availability; and adherence/completion of PEP regimens was moderate to low, impeded by side effects and a belief that completing regimens was unnecessary to avert seroconversion. HCWs consistently expressed concern about HIV stigma.

**Discussion:**

Findings are limited by the inconsistent use of constructs across studies and a lack of clarity about reporting exposure events. Multi‐level approaches are needed to address the interplay of individual, social and structural barriers that diminish HCWs’ PEP use. Improved training, incident reporting, 24‐hour access to non‐stigmatizing PEP services and monitoring of adherence/completion are essential to optimizing HCWs’ PEP use.

**Conclusions:**

Lessons from HCWs’ experience in LMICs may inform understanding of PEP under‐use among people in these settings with non‐occupational exposures.

## 1 INTRODUCTION

Over the 40‐year HIV pandemic, significant progress has been made in reducing HIV incidence, expanding access to life‐saving medications, and providing care and support to people affected by HIV and AIDS globally. This has produced optimism that the HIV pandemic can be halted in the coming decades, leading to global and national plans to end HIV as a public health threat [1, 2]. Much of this optimism derives from developing new technologies for treatment and prevention, chiefly based on highly active antiretroviral (ARV) drugs. Combination antiretroviral therapy (ART) taken as prescribed by people with HIV not only improves their health and lifespan but also prevents them from transmitting HIV sexually to others [3]. The remarkable expansion of ART delivery through such mechanisms as the U.S. President's Emergency Plan for AIDS Relief (PEPFAR) and the Global Fund to Fight AIDS, Tuberculosis, and Malaria (Global Fund), has resulted in about 75% of all people living with HIV globally having access to ART, and approximately 68% of all people living with HIV being virally suppressed (although this proportion varies greatly by population group, region, and sex and gender) [2, 4]. ARVs taken as pre‐exposure prophylaxis (PrEP) by people without HIV significantly reduce their chances of acquiring the virus when an exposure occurs [5]. To date, however, global expansion and uptake of ARVs for primary HIV prevention has not scaled as rapidly as ART for treatment, in part due to a lack of sustainable programmes, suboptimal prevention prioritization and resource allocation, and other structural barriers limiting access [6, 7, 8]. Post‐exposure prophylaxis (PEP), with ARVs taken shortly after needlestick injuries (NSIs) or other exposure to infected blood or bodily fluids, is also effective in preventing seroconversion [9, 10].

Notwithstanding the existence of these highly efficacious ARV‐based strategies for preventing both transmission and acquisition of HIV, in addition to other prevention methods (e.g. medical male circumcision, condom promotion, behaviour change, etc.), UNAIDS has warned that “progress in reducing HIV incidence globally has slowed significantly rather than accelerating, as required to stop the pandemic.” [11] While meaningful declines in HIV incidence have occurred in the Caribbean and western Africa, increases have occurred in 38 countries since 2015 [2]. In 2022, although the estimated 1.3 million new cases of HIV suggested strong declines in high‐burden regions, they still exceeded global targets by nearly 1 million [12].

The ongoing challenges of curbing HIV, particularly in regions with increasing numbers of cases, highlight the urgent need for improved and sustained access, uptake and adherence to effective prevention and treatment services as part of a comprehensive response. A great deal of research has identified related barriers that are influenced by many context‐specific behavioural, social and structural factors [13]. A better understanding of the operations of these factors is key to collective efforts to end the HIV pandemic [14, 15].

A prime example of the underutilization of an efficacious HIV prevention technology, even when it is accessible and usually provided at no cost to the user, is PEP among healthcare workers (HCWs). While the data supporting PEP efficacy derive from animal models and observational studies among humans, not randomized controlled trials, they have provided sufficient biological plausibility to establish PEP as a standard HIV prevention method [16]. In the UK, a retrospective case‐control study among HCWs demonstrated an 80% reduction in HIV infections with PEP; and national surveillance data from 1997 to 2018 reported no new HIV infections among HCWs who received PEP within 72 hours of exposure [9, 17].

In practice, PEP is recommended to HCWs when there is a known or potential HIV exposure at the workplace, historically referred to as “occupational exposure.” According to World Health Organization (WHO) guidance, healthcare facilities should have formal policies and procedures for both preventing and mitigating such exposures, including the promotion of universal precautions, safe injection practices, incident reporting, real‐time information on HIV status of index cases and immediate access to testing, ARV regimens, and follow‐up care [18]. These practices are informed by global guidance documents on PEP for occupational exposures that were initially issued by the WHO in 1998 and subsequently updated in 2014, as well as by national PEP guidelines [19]. Despite the existence of guidance and workplace policies, PEP use among HCWs remains uneven globally [20, 21]. A recent study modelled the effects of high uptake of a three‐drug regimen of PEP in African communities. It concluded such uptake would be sufficient to reduce HIV incidence by 31% over 20 years and would be cost‐effective and cost‐saving [22].

To better understand the dynamics underlying HCWs’ use or non‐use of occupational PEP, we conducted a scoping review of the literature about HCWs’ perceptions, attitudes, priorities, needs and experiences related to PEP use for HIV prevention. The purpose of this review was to better understand the behavioural, social and structural factors facilitating and impeding PEP use among HCWs to inform interventions to further protect HCWs’ health and wellbeing. Knowledge about PEP use dynamics for HCWs also can inform efforts to optimize HIV prevention and reduce new HIV cases globally for anyone exposed to HIV.

## 2 METHODS

This study is part of a larger scoping review of literature on values and preferences for PEP use published from 2014 through 2022 that was conducted to provide background for informal stakeholder consultation to prepare for updates to the WHO's 2014 global PEP guidance, co‐hosted by the WHO and the Bill & Melinda Gates Foundation (BMGF) in early 2023. For the original study, a search was conducted through PubMed, PsycInfo and Google Scholar using various search terms: *HIV, postexposure/post‐exposure prophylaxis, acceptability*, and *values and preferences*. The rationale for the start date of the search was to identify articles written after the 2014 publication of the WHO guidance; and the end date was determined by proximity to the WHO‐BMGF consultation. The search, conducted between November 2022 and January 2023, was run until saturation was achieved (meaning the same articles repeatedly appeared through multiple searches combining terms differently) and yielded 331 studies of potential relevance. To be included in the scoping review, studies had to: be full‐text articles or letters to the editor with data, be published in peer‐reviewed literature, describe the study design and present quantitative or qualitative data. One hundred and fifty‐nine studies published in medical, global health, HIV and social science journals met inclusion criteria.

We subsequently performed a similar, secondary keyword search of the titles and abstracts of the articles to identify those that specifically addressed factors influencing PEP use among HCWs with occupational HIV exposures, using the terms *healthcare workers* and *occupational exposure*. This yielded a sample of 53 scientific articles (Table 1) published in a wide variety of journals, including a number from low‐ and middle‐income countries (LMICs). Two authors (JDA, ADF) undertook a narrative review of the studies using established scoping review methodology [23] to assess factors influencing PEP use among HCWs in diverse geographic locations and workplace institutions. Consistent with scoping review methods, which may not use formal coding instruments common to qualitative and quantitative analyses, we inductively identified themes from the titles and contents of the articles, comparing them to reach a consensus.

**Table 1:** Studies of post‐exposure prophylaxis (PEP) among healthcare workers (HCWs), 2014−2022

Author and year	Country	Sample (*N*)	Age (years)	% Male	% Female	HCW type	Setting	Aware	Accept	Avail	Uptake	Adhere	Main findings
Adebimpe, 2018 [36]	Nigeria	300	>25	33.7	66.3	RN, CH, ML	Government Health Facilities	Y	Y	Y	Y	N	60.3% had heard of PEP, 2.7% had good knowledge, 13.3% knew correct drug combinations, 14.7% had needlestick injuries, 65.9% of whom used PEP, 81.2% aware of national guidelines
Ademe et al., 2020 [74]	Ethiopia	422	26−30	58.1	41.9	RN	Referral Hospitals	Y	Y	N	N	N/A	72.5% had good knowledge; 75.2% had a positive attitude; 67.1% had exposure to sharp injuries
Aigbodion et al., 2019 [69]	South Africa	175	24−30	44.6	55.4	MD	Major/Tertiary Hospitals	Y	N/A	N/A	Y	Y	77.7% exposed, 77.5% initiated PEP, 63.8% completed PEP, 1.1% acquired HIV
Ajibola et al., 2014 [28]	Nigeria	300	24−30	30.3	69.7	MD, RN	Academic Health Centres	Y	Y	N/A	Y	Y	83.3% aware of PEP, 54% knew PEP timing, 15.3% knew PEP duration, 6.3% used PEP
Aminde et al., 2015 [38]	Cameroon	154	∼23	57.8	42.2	MS	Academic Health Centres	Y	N/A	Y	Y	N	89% aware of PEP, 61.7% moderate knowledge, 35.1% knew PEP duration, 4.9% used PEP
Angadi et al., 2016 [25]	India	150	N/A	56.0	44.0	MD	Hospitals and Health Centres	Y	Y	N/A	Y	Y	92.4% lifetime NSI prevalence; 100% aware of HIV‐PEP; 67% received PEP; 38% completed PEP
Anozie et al., 2016 [75]	Nigeria	68	20–50	17.6	82.4	HJ	Major/Tertiary Hospitals	Y	N/A	N/A	N/A	N/A	50% aware of PEP
Anteneh et al., 2019 [44]	Ethiopia	220	∼21−26	62.7	37.3	MS	Academic Health Centres	Y	Y	N/A	Y	Y	29.5% had adequate knowledge, 90% had a positive attitude, 16.8% needed PEP, 8.1% took PEP
Babanawo et al., 2018 [27]	Ghana	185	20−59	28.6	71.4	HW	Referral Hospitals	Y	Y	Y	Y	N	96.8% at risk of occupational exposure, 83.2% reported exposure, 97.3% knew about PEP, 90.8% acknowledged PEP's effectiveness
Bakshi et al., 2015 [76]	India	600	18−22	36.5	63.5	MS	Academic Health Centres	Y	N/A	N/A	N/A	N/A	57.9% awareness of PEP
Bareki and Tenego, 2018 [70]	Botswana	199	20−30	42.9	57.1	MD, RN	Referral Hospitals	Y	Y	N/A	Y	Y	97.4% knew about PEP, 84.5% believed in PEP's effectiveness, 53.7% exposed to risky conditions, 74.8% took PEP
Beyera and Chercos, 2015 [59]	Ethiopia	162	∼23−35	63.0	37.0	HW	Hospitals and Health Centres	Y	N/A	N/A	Y	N/A	25.3% took PEP; reasons for not taking included negligence and lack of awareness
Courtenay‐Quirk et al., 2016 [62]	Botswana, Tanzania and Zambia	3298	N/A	N/A	N/A	HW	Government Health Facilities	N/A	N/A	N/A	N/A	N/A	62.9% reported an exposure; 39% in the past 6 months; 35.6% reported them; 10.6% did not know where to report
Dayyab et al., 2018 [68]	Nigeria	70	N/A	N/A	N/A	HW	Major/Tertiary Hospitals	Y	N/A	Y	Y	Y	73% started PEP, 27% discontinued it
Degavi et al., 2020 [50]	Ethiopia	306	22−57	40.0	60.0	RN	Hospitals and Health Centres	Y	N/A	Y	Y	Y	24.3% used PEP, 91.9% heard about PEP, 77.7% completed PEP
Dhital et al., 2017 [77]	Nepal	50	20−35	N/A	N/A	RN	Referral Hospitals	Y	N/A	N/A	N	N/A	66% knew about PEP; 39.39% knew first aid for needle prick; 60% knew the best time to start PEP; 56% knew the HIV test schedule after exposure
Dulcie et al., 2017 [31]	India	200	N/A	48.0	52.0	MS	Academic Health Centres	Y	Y	N/A	Y	N	87% heard of PEP, 15.5% had formal training, 40.5% knew the ideal PEP regimen, 44% knew the correct drugs, 98% considered PEP important, 89% felt at risk, 23% had exposure, 45.7% received PEP
Ekundayo and Ogbaini‐Emovon, 2014 [37]	Nigeria	187	26−50	72.2	27.8	MD	Academic Health Centres	Y	Y	Y	Y	N	66% had good knowledge, 95% would recommend PEP, 85% would take PEP if exposed, 10.2% had accidental exposure, 47.4% of exposed took PEP
Eticha and Gemeda, 2019 [45]	Ethiopia	311	20−30	50.5	49.5	MD, RN, ML, MW	Academic Health Centres	Y	Y	Y	Y	Y	83% had adequate knowledge of PEP, 43.4% had unfavourable attitude, 17% were exposed to HIV, 71.7% took PEP, 44.8% completed PEP
Gebreslase and Burah, 2014 [39]	Ethiopia	190	N/A	N/A	N/A	HW	Government Health Facilities	Y	N/A	N/A	Y	Y	19.6% used PEP, the main reasons for not using PEP included negative HIV status of the source and negligence, 80% completed PEP
Gupta et al., 2015 [51]	India	4057	N/A	N/A	N/A	HW	Government Health Facilities	Y	N/A	Y	Y	Y	1450 HCWs suffered from occupational exposures, 15% started on PEP, 98% completed full course
Iliyasu et al., 2020 [42]	Nigeria	273	N/A	N/A	N/A	MS	Academic Health Centres	Y	Y	N/A	Y	Y	98.2% heard of PEP; 26% had adequate knowledge; 27.8% reported accidental exposure; 13% of exposed received PEP
Kabotho and Chivese, 2020 [29]	South Africa	160	N/A	N/A	N/A	RN	Major/Tertiary Hospitals	Y	Y	N	Y	Y	Positive attitude towards HIV PEP; sub‐optimal knowledge and uptake of PEP
Kabyemera et al., 2015 [60]	Tanzania	236	>40	N/A	N/A	HW	N/A	Y	N/A	Yes	Y	N/A	31% needlestick injuries, 26% splashes, 28% of splashes reported, 80% needle sticks reported, 39% received PEP
Kassa et al., 2016	Botswana	1624	∼21−39	28.0	72.0	MD, RN, LW	Government Health Facilities	Y	Y	Y	Y	Y	67% received BPE management training, 37% reported exposure, 69% of those received PEP, 71% completed PEP
Khadgi et al., 2022 [34]	Nepal	130	∼20−25	N/A	N/A	RN	Hospitals and Health Centres	Y	N	N	N	N/A	99.3% knew about PEP; 35.8% had adequate knowledge; no history of occupational exposure reported
Kimaro et al., 2018 [54]	Tanzania	239	∼28−50	38.1	61.9	HW	Hospitals and Health Centres	Y	N	Y	Y	Y	52% had inadequate knowledge of PEP, 50.6% experienced occupational exposure, 26.4% started PEP, 23.1% completed PEP
Mabwe et al., 2017 [55]	Tanzania	221	N/A	N/A	N/A	HW	Hospitals and Health Centres	Y	N/A	Y	Y	N/A	27.1% experienced exposure, 11.7% used PEP, awareness of PEP was a significant predictor of its use
Makhado and Davhana‐Maselesele, 2016 [64]	South Africa	240	N/A	N/A	N/A	RN	Major/Tertiary Hospitals	Y	Y	Y	Y	N/A	40% unaware of PEP, 22% unsure of availability, 29% sought PEP
Mashoto et al., 2015 [61]	Tanzania	401	N/A	N/A	N/A	HW	Major/Tertiary Hospitals	Y	N/A	Y	N	N/A	96.3% at risk of exposure, 71.6% knew contact for exposure
Mill et al., 2014 [78]	Uganda	16	36−48	N/A	N/A	RN	Academic Health Centres	Y	Y	N/A	N	N/A	Common needlestick injuries, varying PEP knowledge, some reluctance to report due to stigma and side effects concerns
Mponela et al., 2015 [79]	Tanzania	291	∼42	29.2	70.8	HW	Hospitals and Health Centres	Y	N/A	Y	Y	N/A	35.1% had occupational exposure, 22.5% used PEP, and knowledge and reporting of exposure were significant predictors of PEP use
Mulatu, 2019 [67]	Ethiopia	114	∼21−35	43.9	56.1	MD, RN, MW, LT	Hospitals and Health Centres	Y	N/A	N/A	Y	Y	6.9% utilized PEP, training on PEP significantly increased utilization, three (60%) had completed taking PEP
Mushambi et al., 2021 [57]	Zimbabwe	154	N/A	N/A	N/A	MD, HW	Hospitals and Health Centres	Y	N/A	Y	Y	Y	96% started PEP, 11% completed the 28‐day course, low follow‐up HIV testing
Ncube et al., 2014 [80]	South Africa	169	∼20	24.0	76.0	MS	Academic Health Centres	Y	Y	N/A	N	N/A	28% reported knowledge of NO‐PEP, 67% heard about it from lecturers, 89% recognized the benefits of learning about NO‐PEP
Ngwa et al., 2018 [26]	Cameroon	216	N/A	N/A	N/A	HW	Referral Hospitals	Y	Y	N	Y	N	58% had low knowledge, 61% positive attitude, 51% exposed, 19.1% uptake among exposed
Oche et al., 2018 [46]	Nigeria	156	∼30−39	61.5	38.5	HW	Academic Health Centres	Y	Y	N	Y	N	87.2% heard of PEP, 71.2% good knowledge, 86.8% positive attitude towards PEP
Odinaka et al., 2016 [58]	Nigeria	150	N/A	46.0	54.0	MD	Government Health Facilities	Y	N/A	N/A	Y	Y	96% were aware of PEP, 46.5% mean knowledge score and 60.7% had occupational exposure; only 10 (11%) received PEP, with only seven (7.7%) of them completing the PEP for 4 weeks
Okoh and Saheeb, 2016 [66]	Nigeria	54	∼31−40	74.1	25.9	MD	Academic Health Centres	Y	Y	N/A	Y	N	68.5% inadequate knowledge of PEP, 81.5% positive attitude towards PEP, 46.3% exposed to HIV/HBV risky conditions, 36% took PEP, non‐use due to fear/stigma (94%)
Onuoha and Omosivie, 2019 [30]	Nigeria	199	20−59	35.2	64.8	MD, RN, LS	HIV Treatment Centres	Y	Y	Y	Y	N	39.7% had good knowledge, 96.5% had a positive attitude towards PEP, 22.1% had possible exposure, 45.5% of those exposed took PEP
Patel et al., 2018 [32]	India	450	∼20−24	53.8	46.2	MS	Academic Health Centres	Y	N/A	N/A	N	N/A	90.22% aware of diseases transmitted by NSI, 86.22% believed PEP is important, 49.11% had undergone training on NSI and PEP
Rasweswe et al., 2020 [41]	South Africa	94	18−59	23.0	77.0	RN	Major/Tertiary Hospitals	Y	N/A	Y	N	N/A	90.43% had heard about HIV PEP, 84% did not know where to access HIV PEP, 55.32% were unaware of HIV PEP guidelines
Sendo, 2014 [40]	Ethiopia	185	∼20−23	47.6	52.4	NS	Academic Health Centres	Y	N/A	N/A	Y	N	67.1% heard about PEP, 29.2% were exposed to HIV risk, 59.3% of those exposed started PEP
Shamil et al., 2021 [47]	Ethiopia	217	20−30	51.6	48.4	MD, RN, LT, PH, MW	Healthcare Facilities	Y	Y	N	Y	N	35% had inadequate knowledge, 32.3% had unfavourable attitude, 68.4% of exposed tried to get PEP and 90.7% of those who tried to get PEP received it
Singh et al., 2015 [33]	Pakistan	609	20−45	48.8	51.2	MD, RN, LT, HT	Government Health Facilities	Y	N/A	N/A	Y	N	53.4% had heard of PEP, 27.6% believe PEP should be taken after any needlestick injury, 50.4% attended training on PEP, 35.3% of the respondents were placed on PEP after exposure
Singh et al., 2015 [33]	India	220	20−40	38.6	61.4	HW	Major/Tertiary Hospitals	Y	Y	N/A	Y	N	65.5% heard of PEP, 45% knew when to initiate PEP, 23.2% knew the maximum delay, 52.7% knew PEP duration, 21.4% were exposed, 14.9% of exposed took PEP
Suglo et al., 2021 [52]	Ghana	199	∼25−39	30.7	69.3	HW	Academic Health Centres	Y	N/A	N/A	Y	Y	17.9% adhered to PEP, 90.1% were aware of PEP, 44.4% took PEP after exposure
Tesfaye et al., 2014 [48]	Ethiopia	72	∼25−34	43.1	56.9	HW	Hospitals and Health Centres	Y	Y	N	Y	N	100% heard about HIV PEP, 54.2% knew the correct drugs for PEP, 55.6% knew the initiation time for PEP, 16.7% of those exposed used PEP
Tetteh et al., 2015 [53]	Ghana	295	N/A	40.7	59.3	HW	Academic Health Centres	Y	N/A	Y	Y	Y	60.0% occurred during needle disposal, 24.6% during cannula insertion and 62.6% completed PEP with no seroconversions
Tetteh et al., 2015 [53]	Ghana	228	18−50	43	59.9	HW, MS	Academic Health Centres	N	N/A	Y	Y	Y	80% reported exposures, 77% adhered to completion (higher for shorter regimens and fewer adverse events)
Tshering et al., 2020 [35]	Bhutan	221	22−42	43.4	56.6	RN	Referral Hospitals	Y	Y	Y	Y	Y	51.1% heard about PEP, 80.1% had poor knowledge, 92.3% positive attitude, 43% exposed to HIV risk, 2.1% took PEP
Uzochukwu et al., 2014 [49]	Nigeria	129	∼31−45	13.2	86.8	RN	Hospitals and Health Centres	Y	Y	Y	Y	N	86% knew PEP, 92.2% agreed PEP reduces HIV risk, 29% treated with PEP on exposure
Vardhini et al., 2020 [63]	India	405	21−44	25.1	74.9	RN, PW	Major/Tertiary Hospitals	Y	N	N	Y	N	Nurses had higher knowledge (71.1%) and uptake (23%) compared to paramedical workers (36.3% and 14.3%)

***Notes***: Studies assessed: Aware = PEP awareness and knowledge; Accept = PEP acceptability; Avail = PEP accessibility and availability; Uptake = PEP uptake and use; Adhere = PEP adherence and regimen completion.

Abbreviations: CH, community health worker; HCW, healthcare worker; HJ, hospital janitorial staff; HT, health technician; HW, healthcare worker—no further specification; LT, lab technician; LW, lab worker; MD, medical doctor/physician; ML, medical lab scientist/technician; MS, medical student; MW, midwife; N, no; N/A, not available—the study did not explicitly discuss that aspect; NS, nursing student; PH, public health officer; PW, paramedical worker; RN, registered nurse; Y, yes.

## 3 RESULTS

All but two studies were conducted in LMICs in Africa and Asia. As such, our analysis focuses on findings from these LMICs. We found that PEP use among HCWs in these settings is influenced by behavioural, social and structural influences that manifest along a trajectory of PEP engagement. The domains of this trajectory, which emerged as thematic categories, included: Awareness and Knowledge; Acceptability; Access and Availability; Uptake and Use; and Adherence and Completion. It is worth noting that these domains sometimes overlap, and many studies examined more than one of them at the same time, as detailed below.

### 3.1 Awareness and knowledge

A first step towards using PEP is being aware that it exists and having knowledge of how it works to prevent HIV acquisition. HCWs are in a better position than most other populations to be aware of PEP and know the specifics of how it works because of their training as providers at potential risk for occupational exposure to HIV and other blood‐borne infections. But the studies we reviewed that assessed awareness and knowledge found a great deal of variation, particularly in levels of knowledge.

The terms “awareness” and “knowledge” are often used interchangeably in public health research but in the studies we reviewed, both were assessed as separate constructs [24]. Most studies included information about the definitions and measures used for each construct: for awareness, these were variations of having heard of PEP; and for knowledge, they were understanding of HIV transmission, PEP as a means of prevention following exposure, timeframes for initiating PEP (within 72 hours of exposure), ARV drug regimens (2−3 drugs) and regimen course (28 days). Many of these studies combined assessments of awareness, knowledge, attitudes and use—often collectively referred to as “practice” of PEP for exposed HCWs. In these cases, attitudes typically were measured by a belief that PEP is a good strategy; and practice was measured by exposure experience, exposure reporting, and, where included, PEP uptake, use, adherence and completion.

Studies that addressed awareness and knowledge were conducted in African and Asian countries, with one in the United States, and research settings included urban and rural hospitals, clinics, emergency departments, teaching and tertiary institutions, and medical (including pharmacy, dental and nursing) schools. Across occupations, institutional settings and geographic locales, HCWs and those training to become them displayed high levels of awareness of PEP for HIV prevention in the context of occupational exposures, ranging from 28% to 100% [25, 26].

The same was not true for knowledge of how PEP works. Multiple studies found that low to moderate rates of complete PEP knowledge were common, even where awareness of PEP was high. For example, a study of 185 HCWs (doctors, nurses, midwives and laboratory technicians) in a regional hospital in Koforidua, Ghana found that 97.3% were aware of PEP, 90.8% acknowledged its effectiveness in preventing HIV acquisition and 93% knew PEP should be initiated within 72 hours of exposure; however, only 3.9% knew that the treatment course was 28 days and only 25.1% knew the correct drug regimen [27]. Similarly, in a teaching hospital in Lagos, Nigeria, 83% of the 154 study participants from various clinical specialties were aware of PEP. Still, only 54% knew when to initiate it following exposure, and 32% could name at least two of the recommended drugs in the regimen [28]. This low‐ to moderate‐level PEP knowledge was found among diverse HCWs and medical students in other African countries [29, 30] as well as in India [31, 32, 33], Nepal [34], Pakistan [33] and Bhutan [35].

Some authors attributed low PEP knowledge to limited education and training about national guidelines and institutional protocols [36, 37]. In some cases, HCWs did not know to whom to report a potential (or known) occupational exposure, which stymied their access to PEP [28, 38]. In other cases, HCWs were not aware of current PEP policies in their institutions [27, 37, 39, 40]. For example, 84% of nurses in a tertiary hospital in Gauteng province, South Africa—a locale with stable, relatively high HIV prevalence (meaning an elevated risk of occupational exposure)—did not know where to access PEP; 55% were unaware of any HIV PEP guidelines used in their hospital [41]. Other studies showed that higher PEP knowledge is associated with higher levels of education and more years of experience [38, 42]. Few studies provided information about the PEP training HCWs receive, so it is difficult to make any conclusions about the specific nature of the gaps.

### 3.2 Acceptability

One may have knowledge about PEP but choose not to act on it, if one does not find PEP acceptable, which we defined as: “a multi‐faceted construct that reflects the extent to which people delivering or receiving a healthcare intervention consider it to be appropriate, based on anticipated or experienced cognitive and emotional responses to the intervention” [43]. This definition acknowledges that acceptability is influenced by a range of factors, including attitudes towards PEP, its perceived effectiveness, burden, ethicality, alignment with personal values and beliefs, opportunity costs and one's self‐efficacy to use it properly.

Studies that assessed PEP's acceptability among HCWs that fit this definition usually operationalized it as “willing to accept” or “had favourable attitudes” towards PEP. The study samples included physicians, nurses and medical students working in urban and rural hospitals, clinics and health centres in Africa and Asia. Overall, PEP acceptability was moderate to high, ranging from 61% to 92% [26, 29, 30, 44, 45, 46, 47, 48, 49]. For example, a study in a Lagos Teaching Hospital found that 73% of HCWs were willing to accept PEP if the need arose [28]. Similarly, a study of nurses in Bhutan found that 92% had favourable attitudes towards PEP [35]. Interestingly, favourable attitudes about PEP did not necessarily increase its use. On the one hand, a study of HCWs at a teaching hospital in Sokoto, Nigeria found that those with positive attitudes about PEP and who found it acceptable in cases of occupational exposure were more likely to use it, even if they had gaps in knowledge about its dosage, regimens and effectiveness [46]. On the other hand, a study among HCWs at a hospital in Gimbi, Ethiopia found no association between PEP attitudes and practice [48].

### 3.3 Access and availability

If PEP is deemed acceptable to an HCW, its use is affected by its accessibility and availability. These terms have distinct meanings: “access” refers to the ease of reaching or obtaining something. In contrast “availability” refers to the presence or readiness of something, but they are related and are sometimes used interchangeably. Insights about PEP access and availability among HCWs, including nurses, other primary healthcare providers and medical students, came from studies predominantly (94%) conducted in Africa [29, 30, 35, 38, 44, 45, 46, 50, 51, 52, 53]. We report on the relevant studies according to the terms—access and/or availability—used in each.

The perceived accessibility of PEP following occupational HIV exposure varied widely among HCWs. In one instance, over 70% of exposed respondents took PEP, implying its high accessibility [45], while in others, these services were accessible only to approximately 20% of respondents [54, 55]. In several studies, a lack of training and access to relevant resources were pronounced. For example, 84% of South African nurses surveyed did not know where to access PEP in their institutions, and 55% were unaware of related guidelines [41]. In some settings, the proportion of exposed HCWs taking PEP was alarmingly low. A study of Bhutanese nurses found that only 2.1% of those with occupational exposures had taken PEP, due to a lack of access to related services and insufficient support for reporting incidents [35]. A study of primary HCWs in Enugu State, Nigeria, found that 95.3% of HCWs reported PEP as inaccessible [49]. These findings are particularly concerning in light of the fact that there is clear evidence that perceived programme access is an important determinant of PEP utilization, even in the face of such barriers as long wait times and uneven awareness of related services [53].

Several studies underscored the inconsistency and, in many cases, the inadequacy of PEP availability for HCWs following occupational HIV exposures. In Southwestern Nigeria, a study found that 25% of HCWs did not use PEP following NSIs because it was unavailable [36]. Similarly, studies in Ghana and Bhutan showed that the absence of PEP services and uncertainty about their availability accounted for why many HCWs did not report exposures or take ARVs [35, 52]. In other instances, a lack of knowledge about the availability of PEP protocols and services was pronounced. For example, an estimated 16.9% of exposed Cameroonian medical students were unaware of their hospital's PEP protocol [38], and nearly 60% of Ethiopian medical students were unaware of their facilities’ PEP guidelines [44].

Further, many study participants in locations where PEP services and medicines were available at all hours of the day were unaware of this. A study of Ethiopian HCWs noted that only 40.5% of respondents knew that PEP services were available 24 hours a day; nearly 60% were unaware that the service existed [50]. In Nigeria, only 44.9% of HCWs expressed knowledge of a PEP protocol in their workplace [46]. A 2010 study of Indian HCWs found that only 46% of hospitals confirmed 24‐hour‐a‐day availability of PEP medicines, and only 37% had clear PEP reporting mechanisms [56]. Taken together, these findings are concerning because limited PEP access and availability may increase the probability of HIV seroconversion among HCWs following occupational exposures. In addition, inadequate access, combined with insufficient knowledge and awareness of the benefits of PEP, may discourage incident reporting and further reduce the utilization of a safe and effective HIV prevention measure. These shortcomings also may increase stress and anxiety among HCWs and worsen HIV‐related stigma, which may have downstream effects on healthcare access and delivery.

### 3.4 Uptake and use

PEP may be accessible and available to HCWs, but that does not ensure its uptake—meaning the initiation of a PEP drug regimen following occupational exposure—and use—meaning taking more than one dose of PEP over the timeframe of the prescription. Across African and Asian studies involving various HCW types and settings, PEP uptake and use—terms often used interchangeably—were relatively low. The percentage of HCWs who took up PEP ranged from 2.1% among those in a national referral hospital in Bhutan [35] to 96.0% among those at the Parirenyatwa Group of Hospitals in Harare, Zimbabwe [57].

A first step in accessing and initiating PEP after a known or potential HIV exposure is to report the event to the relevant persons or entities in the healthcare setting. We attempted to ascertain what proportion of HCWs did so and then see how many of those took up PEP. Unfortunately, the authors of many studies of PEP uptake and use that refer to HCWs who “report an exposure” did not clarify if they meant that the HCWs *reported their exposure to the healthcare system at the time the exposure took place* or if they meant that the HCWs *reported to the study team that they had experienced an exposure at some time*. (We discuss this further in the Limitations section below.) Whichever way “report an exposure” was meant, studies found consistently that HCWs with occupational exposures had low uptake of PEP.

For example, a study of 199 frontline HCWs in a teaching hospital in Ghana found that 58.8% had experienced either an NSI or a blood splash while at work. But 51.3% of these did not report their exposure at the time, citing not knowing to whom to report, being unaware of the availability of PEP and/or negative HIV test for the source. Of those who reported their exposure, 44.4% received PEP [52]. A study of 150 paediatricians attending an annual conference in Nigeria found that 60.7% had occupational exposure, but only 11% received PEP [58].

Many studies did not report whether the HCWs knew the HIV serostatus of the index patient; but even when the answer was “yes,” uptake of PEP did not always follow. For example, in a study of 221 nurses at a national referral hospital in Bhutan, 67.4% of the 95 respondents who reported being exposed said they confirmed the HIV status of the relevant patients, 40.6% of whom were living with HIV. Still, only 2.1% of the HIV‐exposed HCW received PEP [35]. Among HCWs in one referral hospital and three health centres in northwest Ethiopia, only 25.3% utilized PEP even with known exposure [59]. Notably, at an HIV treatment unit in Nigeria—that is where patients by definition had HIV—only 45% of HCWs with possible exposures took PEP [30].

Study participants provided several reasons for not reporting exposure to the relevant persons or entities in their workplace. These included fear of stigma and discrimination [60]; not knowing to whom they should report an exposure [28, 61]; lack of support and encouragement to report exposure [26, 35, 40]; lack of awareness of PEP service and protocol [40, 59]; belief in low‐risk exposure [62, 63]; and being “afraid to go through the whole process”—of reporting exposure, taking PEP drugs and taking an HIV test [64].

Reasons for not taking up and using PEP included fear of stigma and discrimination [47, 58, 65, 66]; fear of side effects of the PEP drugs [30, 35, 40, 47, 58]; lack of information about the existence of services [47]; the source patient tested HIV negative [66]; ignorance about PEP [36]; a belief that taking PEP was not important [35]; a belief that the person could not acquire HIV [36]; and a lack of availability of PEP service and/or drug(s) [49]. Although it was not mentioned in the studies, it is also possible that HCWs did not take up PEP because they already were living with HIV.

A few studies looked at factors that were positively associated with PEP uptake, which included having had training on PEP [39, 67]; being of female sex/gender [59]; having reported the exposure at the time it occurred [55, 59]; knowing the serostatus of the source patient [55]; and knowing that PEP was available [30, 55].

As would be expected, in many of the studies reviewed, PEP uptake and use among occupationally exposed HCWs frequently overlapped with awareness and knowledge and with adherence and completion. However, as previously noted, awareness and knowledge did not necessarily lead to uptake and use, and, similarly, initial uptake and use did not necessarily lead to adherence and completion.

### 3.5 Adherence and completion

The terms “adherence” and “completion” are both related to following and finishing the prescribed course of PEP, which ultimately determines PEP's efficacy. But, theoretically, one can “adhere” to a PEP regimen up to a point, but then not complete the prescription; and one can “complete” the prescription but not strictly adhere to the regimen for taking the drugs. The studies in our review that addressed PEP adherence and completion among HCWs variously used the terms “adherence” and “completion” without defining them. As such, in the following discussion, we use the terms used by the authors to describe their findings. The overall finding from these studies—conducted in African and Asian countries—is that, even when HCWs initiated the use of PEP, they often did not complete the regimen. The PEP adherence and completion levels among those HCWs who began the regimen ranged from a low of 7.7% [58] to a high of 100% [42]. The most salient explanations for HCWs’ PEP non‐completion related to difficulties with the drug regimen—specifically, the types of drugs, their side effects and the 28‐day length of the PEP regimen. For example, a study of 51 HCWs in northwest Nigeria who had a known exposure to a person living with HIV and who initiated PEP found that non‐completion was 2.6 times more likely among those on a non‐tenofovir‐containing regimen than those on tenofovir‐containing regimens, which may be due to experiencing greater side effects with these drugs. Additionally, HCWs prescribed 3 pills per day were more likely to not complete their regimen than those prescribed only 2 pills [68].

A study of 228 exposed HCWs in a teaching hospital in Ghana found that reporting of adverse events (AEs) and adherence to PEP varied according to the drug regimen [53]. The frequency of reporting AEs was 28% among HCWs taking 3TC/AZT for 3 days, 91% for those taking 3TC/AZT for 28 days and 96% for those taking 3TC/AZT/LPV‐RTV for 28 days. Nausea was the most commonly reported AE in all groups. Adherence was 100% in the first group, 56% in the second and 62% in the third. HCWs who never reported AE were 57% more likely to adhere to their regimen than others, and all 53 individuals who did not adhere completely cited AE as their reason. Numerous other studies reported the contribution of side effects to PEP non‐completion, although they did not detail the nature of these [30, 39, 44, 69, 70]. Finally, some HCWs believed the full 28‐day PEP course was unnecessary and stopped taking it [70].

What we learn from these studies is that PEP adherence and completion is a highly contingent practice that is affected to a great extent by the nature of the available drug regimens for HCWs and the potential side effects, including nausea, abdominal cramping and headache [8]. This underscores the need for simplified and better‐tolerated PEP regimens [16].

## 4 DISCUSSION

This scoping review sought to elucidate the dynamics underlying HCWs’ use of PEP as these arrayed along identified themes related to constructs in a PEP use continuum: awareness and knowledge, acceptability, access and availability, uptake and use, and adherence to and completion of dosing regimens. In doing so, we aimed to explore how PEP use may be thwarted and how it may be enhanced among HCWs to improve their health and wellbeing.

The review has several limitations. First, the literature is marked by a lack of standardization in its use of key constructs like “acceptability,” which can refer either to general attitudes towards PEP or agreement about its efficacy in averting HIV acquisition. This adds a layer of complexity that would be minimized with greater attention to consensus definitions. Similarly, many studies lacked clarity about what was meant when asking if participants had “reported exposures,” a phrase that could imply alerting supervisors about an exposure or merely stating to a researcher that an encounter with bodily fluids had occurred in the workplace. Second, many studies did not distinguish whether non‐completion of 28‐day regimens was the result of new information (e.g. the exposure source was found not to be living with HIV) or HCWs’ experience of side effects and/or beliefs that taking the full course was unnecessary. Third, the relatively small sample sizes for studies of HCWs who initiate and complete PEP limits confidence in the generalizability of the findings to the larger universe of HCWs. Fourth, the heterogeneity of the participants across studies obviates comparisons about all aspects of PEP use between categories of HCWs and types of occupational settings. Fifth, there was a dearth of studies that included any qualitative information about why HCWs perceived what they perceived and did what they did, which limits full understanding of PEP use and non‐use from HCWs’ point of view. Sixth, most studies focused on barriers to PEP use among HCWs rather than facilitators, so it is difficult to conclude much about what contributes to optimal PEP use among this population. Seventh, we included in our analysis studies published between 2014 and 2022 for reasons described earlier. Although studies have been published since, they primarily focus on PEP guidelines and occupational exposure incidents; only a few address knowledge and attitudes or PEP use [71, 72, 73]. These studies underscore the importance of PEP education and awareness among HCWs; they do not fundamentally change our understanding of the factors in PEP use identified in our review. Finally, our findings may be limited by their reliance on studies published in English.

Notwithstanding these limitations, our review surfaced essential findings about the PEP engagement trajectory among HCWs, particularly in LMICs, that can inform future work. Although there was high awareness among HCWs that PEP is an effective prevention measure against occupational HIV exposure, knowledge about drug regimens, duration of treatment and specific mechanisms of action was consistently low across occupations, locations and settings. Similarly, many HCWs were unaware of their institution's policies and procedures. Acceptability was generally high among HCWs, suggesting favourable attitudes that appear to increase their willingness to use PEP when available. Perceived accessibility and availability varied considerably partly because PEP is inconsistently available across settings. In addition, there is evidence that HCWs have limited information about institutional PEP policies, reporting procedures and services, such as 24‐hour programmes, where they exist, which represent structural barriers to uptake and use that can be remedied easily. Finally, the review found that most HCWs do not complete PEP once initiated. Several reasons for this emerged, including side effects and beliefs that taking the full 28‐day course was unnecessary. In addition, it was difficult to ascertain whether some HCWs discontinued PEP after learning that index cases that prompted PEP initiation were later found not to be living with HIV.

Although additional research is needed to clarify how best to ensure PEP completion in occupationally exposed HCWs, these findings suggest several steps to reduce associated barriers, starting with better training and knowledge of rapid initiation of PEP to avert HIV acquisition. The studies reviewed showed considerable variability in PEP knowledge and the benefits and risks of its use. Quality PEP training programmes may improve awareness of this prevention modality among HCWs in high HIV prevalence settings. Furthermore, PEP protocols and guidelines must be easily accessible to all, and healthcare facilities should use available channels to promote PEP and educate staff about the frequency of NSIs and other exposures in a non‐stigmatizing manner. Additionally, addressing gaps in access and availability may help to ensure that PEP is used following high‐risk exposure events. All healthcare facilities must ensure that PEP is accessible 24 hours per day and accompanied by clear and concise policies and procedures. Creating a more supportive, less stigmatizing environment for managing occupational exposures may also increase HCWs’ reports of such events and increase PEP use. Finally, improving the completion of PEP regimens demands better‐tolerated drug regimens and enhanced adherence counselling and support to anticipate and manage side effects. Although there is a preferred regimen for PEP, alternative regimens would address known barriers such as drug toxicities, medication shortages, adherence and side effects that affect completion rates [19]. (It is not yet known if or when new, long‐acting oral or injectable medications for HIV treatment and prevention that may address some of these issues will be relevant and feasible for PEP.) Facilities should also monitor PEP adherence and completion to identify and address known gaps in its effective use.

Taken together, these steps may help to ensure the safety and wellbeing of HCWs facing the recurring threat of NSIs and other exposure events that can lead to HIV acquisition and the persistence of the pandemic. They also underscore that initiating and completing PEP following occupational exposures is influenced by a complex mix of behavioural, social and structural factors that require a multi‐level approach to improving PEP use among HCWs. The same is likely true for other populations, including those at risk for HIV acquisition as a consequence of unprotected sex, sexual assault, condom breakage or other exposures, for whom additional issues related to knowing about PEP and being able to find and afford it are more pronounced.

## 5 CONCLUSIONS

By better understanding and addressing gaps in and facilitators of awareness, knowledge, acceptability, access, availability, uptake, use, adherence and completion, we can improve PEP use and develop more effective programmes and interventions to protect individuals after occupational or other exposure to HIV.

## COMPETING INTERESTS

The authors have no competing interests to declare.

## AUTHORS’ CONTRIBUTIONS

JDA conceptualized, designed, collected and analysed the data for the larger study from which this review was derived; JDA and ADF conceptualized, collected and analysed the data selected for this review; JDA and ADF drafted the manuscript with input from SM; ADF prepared Table 1. All authors reviewed, edited and approved the final manuscript.

## FUNDING

JDA's work on the study reported in this manuscript was supported by the Bill & Melinda Gates Foundation, contract #INV‐053199.

## Data Availability

Data sharing is not applicable to this article because it is a review of existing, published literature and no new data were generated during the current study.
